# Role of arachidonic acid metabolism in osteosarcoma prognosis by integrating WGCNA and bioinformatics analysis

**DOI:** 10.1186/s12885-024-13278-3

**Published:** 2025-03-12

**Authors:** Yaling Wang, Peichun HSU, Haiyan Hu, Feng Lin, Xiaokang Wei

**Affiliations:** 1 Department of Oncology, Shanghai Eighth People’s Hospital, Shanghai, China; 2 Department of Orthopedics, Shanghai Sixth People’s Hospital Affiliated to Shanghai Jiao Tong University School of Medicine, Shanghai, China; 3 Shanghai Clinical Research Ward (SCRW), Shanghai Sixth People’s Hospital Affiliated to Shanghai Jiao Tong University School of Medicine, Shanghai, China

**Keywords:** Osteosarcoma, Arachidonic acid, Prognosis, Bioinformatics, WGCNA, Biomarker, Immunotherapy response

## Abstract

**Background:**

Osteosarcoma is a rare tumor with poor clinical outcomes. New therapeutic targets are urgently needed. Previous research indicates that genes abnormally expressed in osteosarcoma are significantly involved in the arachidonic acid (AA) metabolic pathway. However, the role of arachidonic acid metabolism-related genes (AAMRGs) in osteosarcoma prognosis remains unknown.

**Methods:**

Osteosarcoma samples from The Cancer Genome Atlas (TCGA) and Gene Expression Omnibus (GEO) databases were classified into high-score and low-score groups based on AAMRGs scores obtained through ssGSEA analysis. The intersecting genes were identified from weighted gene co-expression network analysis (WGCNA), DEGs (osteosarcoma vs. normal) and DE-AAMRGs (high- vs. low-score). An AA metabolism predictive model of the five AAMRGs were established by Cox regression and the LASSO algorithm. Model performance was evaluated using Kaplan-Meier survival and receiver operating characteristic (ROC) curve analysis. In vitro experiments of the AA related biomarkers was validated.

**Results:**

Our study constructed an AAMRGs prognostic signature (CD36, CLDN11, STOM, EPYC, PANX3). K-M analysis indicated that patients in the low-risk group showed superior overall survival to high-risk group (*p*<0.05). ROC curves showed that all AUC values in the prognostic model exceeded 0.76. By ESTIMATE algorithms, we discovered that patients in high-risk groups had lower immune score, stromal score, and estimate score. Correlation analysis showed the strongest positive correlation between STOM and natural killer cells, and the highest negative association between PANX3 and central memory CD8 T cells. An AAMRGs prognostic signature was constructed for osteosarcoma prognosis.

**Conclusion:**

The study suggested that a high level of AAMRGs might serve as a biomarker for poor prognosis in osteosarcoma and offers a potential explanation for the role of cyclooxygenase inhibitors in cancer. The five biomarkers (CD36, CLDN11, EPYC, PANX3, and STOM) were screened to construct an AAMRGs risk model with prognostic value, providing a new reference for the prognosis and treatment of osteosarcoma.

**Supplementary Information:**

The online version contains supplementary material available at 10.1186/s12885-024-13278-3.

## Introduction

Osteosarcoma (OS) is the most aggressive malignant tumor among bone sarcomas, and represents approximately 56% of cases, predominantly afflicting children, adolescents, and young adults [1]. The current gold-standard approach to managing osteosarcoma involves radical surgical resection coupled with neoadjuvant and adjuvant chemotherapy [2]. In addition, radiotherapy and immunotherapy are increasingly contributing to the multimodal treatment of osteosarcoma [3]. Nevertheless, osteosarcoma is characterized by early metastasis, particularly to the lungs, with recurrence rates observed in 50–60% of patients [4]. Despite the fact that comprehensive treatment strategies have significantly enhanced patient outcomes, those with advanced-stage osteosarcoma face limited therapeutic options, and the 5-year survival rate for individuals with metastatic or recurrent disease remains suboptimal, at only 20–30% [5].

Arachidonic Acid (AA) is a polyunsaturated fatty acid crucial for various cellular processes. It can be liberated by Cytosolic phospholipase A2 (cPLA2) and serves as a precursor to a multitude of bioactive metabolites through enzymatic processes involving cyclooxygenase (COX), lipoxygenase (LOX), and cytochrome P450 (CYP450) pathways [6]. These enzymes catalyze the production of bioactive lipid mediators, including prostaglandins (PGs), leukotrienes (LTs), thromboxane (TX) A2, and eicosanoids, which play crucial roles in regulating inflammation, immunity, and tumorigenesis. Emerging evidence suggests that dysregulation of arachidonic acid metabolism contributes significantly to the pathogenesis of various cancers. Dietze et al. [7] suggested that arachidonic acid metabolism promote tumor development and correlates with adverse clinical outcomes in ovarian cancer through influencing the phenotype of tumor-associated macrophages. In breast cancer, AA can reactivate mTOR signaling, foster cancer growth and angiogenesis [8]. However, some studies revealed that AA assumes an anti-tumor role through three mechanisms: facilitating tumor cell ferroptosis induced by ACSL4, enhancing the anti-tumor CD8 + T-cell response, and rendering tumor cells more responsive to checkpoint therapy [9]. Additionally, Roodhart et al. [10] found that AA secreted from adipocytes can promote ovarian cancer chemoresistance in ovarian cancer patients. Consequently, inhibitors targeting enzymes involved in AA metabolic pathways may provide great potential in resolving chemoresistance [11, 12].

Alterations in the expression and activity of AA metabolic enzymes in tumors suggest that AA plays a significant role in tumor progression and aggressiveness. Inhibitors of COX or LOX enzymes, either alone or in combination with standard treatments, could enhance therapeutic efficacy and improve patient outcomes [13–15]. Additionally, modulating downstream signaling pathways activated by eicosanoids may offer new avenues for intervention [16]. Therefore, integration of AA metabolism-related parameters into existing prognostic models could refine risk stratification and enhance the accuracy of outcome predictions for osteosarcoma patients.

The present study aims to unravel the intricate interplay between biomarkers or genetic signatures associated with AA metabolism and osteosarcoma prognosis, seeking to establish a predictive survival model for osteosarcoma by leveraging transcriptomic and single-cell data obtained from public databases. A comprehensive array of bioinformatics techniques, including gene differential expression analysis and Weighted Gene Co-expression Network Analysis (WGCNA), was utilized. Through this investigative approach, we identified five prognostic biomarkers for osteosarcoma intricately linked with AAM. Moreover, the study explored the pathway enrichment of these biomarkers and examined their association with the tumor microenvironment (TME). This analysis provides profound insights into the molecular mechanisms governing the involvement of genes in arachidonic acid metabolism within the context of osteosarcoma. It unlocks novel prognostic biomarkers, therapeutic targets, and predictive models that revolutionize clinical management strategies in treating osteosarcoma.

## Materials and methods

### Source of data

The TCGA-osteosarcoma dataset, derived from the TCGA-Target database (https://portal.gdc.cancer.gov/), contains 88 osteosarcoma samples, of which 85 include survival information. The GSE99671 and GSE21257 datasets were sourced from Gene Expression Omnibus (GEO) database (https://www.ncbi.nlm.nih.gov/gds). Specifically, the GSE99671 dataset (platform GPL20148) comprises RNA-seq data from fresh-frozen tissue of 18 normal and 18 osteosarcoma samples. Meanwhile, the GSE21257 dataset (platform GPL10295) includes the RNA-seq data of tumor tissue from 53 osteosarcoma samples, each accompanied by survival information. A total of 58 AAM related genes (AAMRGs) were obtained from previous study [17].

### Acquisition of differentially expressed genes (DEGs)

DEGs between the osteosarcoma and normal groups were selected in the GSE99671 dataset by using the edgeR package (v 3.36.0) with P value < 0.05 and |log_2_FC| > 0.5. Followed by, the volcano and heatmap were drawn with the ggVolcano software package (v 0.0.2) and ComplexHeatmap package (v 2.14.0), respectively, illustrating the variance of DEGs.

### Key module genes were screened by WGCNA

The AAMRGs score of each sample was obtained using GSVA package (v 1.46.0) with AAMRGs as the background geneset. Using WGCNA to construct co-expression networks (v 1.70-3) [18]. Initially, clustering was performed through the samples to weed out outliers and ensure the accuracy of the analysis. Then, the optimal soft threshold (β) was selected to construct a scale-free network. Immediately next, the clustering dendrogram was acquired by calculating the adjacency and similarity. Division of modules with dynamic tree-cutting algorithm. Immediately following, we evaluated the correlation between each module with osteosarcoma and AAMRGs score, and selected the key modules that were relevant to both the osteosarcoma and AAMRGs score. Lastly, the genes in the key modules were targeted as key module genes for following analyses.

### Acquisition of DE-AAMRGs

The tumor samples were classified into high- and low-score groups by the median AAMRGs score in the GSE99671 dataset. Then, DE-AAMRGs between two differential score groups were selected by using the edgeR package (v 3.36.0) with *P* value < 0.05 and |log_2_FC| > 0.5. Finally, the Upset package (v 1.4.0) was utilized to take intersections of DEGs, key module genes and DE-AAMRGs to obtain intersecting genes.

### Construction of protein-protein interaction (PPI) networks and functional annotation of intersecting genes

The PPI network was constructed via search tool for the retrieval of interacting genes (STRING) database (http://string-db.org) to observe the connections between intersecting genes. Gene Ontology (GO) and Kyoto Encyclopedia of Genes and Genomes (KEGG) enrichment analyses of intersecting genes was executed via DAVID analysis tool (v 6.8) (*P* value < 0.05) (https://david-d.ncifcrf.gov/).

### Construction of prognostic model

The univariate Cox analysis and least absolute selection and shrinkage operator (LASSO) algorithm were conducted via the glmnet package (v 4.0–2) for intersecting genes to acquire biomarkers. Patients in the TCGA-osteosarcoma dataset were classified into high- and low-risk groups according to the optimal threshold of the risk score. Riskscore = $\:\sum\:_{1}^{\text{n}}\text{c}\text{o}\text{e}\text{f}\left(\text{g}\text{e}\text{n}\text{e}\text{i}\right)\text{*}\text{e}\text{x}\text{p}\text{r}\text{e}\text{s}\text{s}\text{i}\text{o}\text{n}\left(\text{g}\text{e}\text{n}\text{e}\text{i}\right)\sum\:_{1}^{\text{n}}\text{c}\text{o}\text{e}\text{f}\left(\text{g}\text{e}\text{n}\text{e}\text{i}\right)\text{*}\text{e}\text{x}\text{p}\text{r}\text{e}\text{s}\text{s}\text{i}\text{o}\text{n}\left(\text{g}\text{e}\text{n}\text{e}\text{i}\right)$. Subsequently, Kaplan-meier (K-M) survival curves were drawn. The survivalROC package (v 1.0.3) was utilized to compute area under curve (AUC) values for receiver operating characteristic (ROC) curves to assess the predictive accuracy of the model. The prognostic model was verified with GSE21257 dataset.

### Constructing a nomogram

Independent prognostic analyses were performed on clinical characteristics and risk score in the TCGA-osteosarcoma dataset. Immediately after, the nomogram was constructed and visualized via RMS package (v 6.2-0). Next, calibration and decision curve analysis (DCA) were plotted to judge the model performance.

### Clinical features correlation analysis

To understand the correlations between risk scores and clinical features, we compared the differences in risk scores with different clinical information. The differential analyses were carried out by the Wilcoxon text method and the results were visualized by violin plots. Then, survival analyses were implemented using the survival package in the differential risk subgroups for different stratified clinical characteristics.

### Functional and annotation analyses and single-gene GSEA analysis

Firstly, the c2.cp.kegg.v7.5.1.symbols.gmt dataset was obtained through Molecular signatures database (MSigDB) (https://www.gsea-msigdb.org/gsea/msigdb) as the background gene set for the enrichment analysis. Then, enrichment scores were computed for each pathway using the GSVA package (v 1.46.0), and the limma package (v 3.54.0) was used to filter out the two risk subgroups between which there were pathways with differences between the two risk subgroups. The single-gene gene set enrichment analysis (GSEA) was performed to find the enriched regulatory pathways and biological functions of biomarkers with NOM p-val < 0.05 and |NES| > 1. The top 10 results for KEGG significance were visualized separately.

### Immune-infiltration analysis

In order to compare whether the immune score, ESTIMATE score and stromal score varied between the differential risk subgroups, we used the ESTIMATE algorithm (v 1.0.13) to calculate these scores in the two risk subgroups and then compared them by the Wilcoxon method. The proportion of 28 immune cell subtypes were computed for each sample by single-sample GSEA (ssGSEA) algorithm. Afterwards, the differential immune cells between two risk subgroups were compared and box-plot was plotted. Meanwhile, the correlation analysis was performed between immune cells and biomarkers using Spearman method.

### Immunotherapy response prediction analysis

Firstly, immune function pathway enrichment scores and tertiary lymphoid structure (TLS)-associated gene feature scores were computed using the ssGSEA algorithm for patients in differential risk subgroups. Then, the correlation between the risk score and the immune pathway enrichment score, the TLS score, and the individual immune checkpoints was computed by the Spearman method, respectively. In addition, the TIDE, Dysfunction, CD274, and Exclusion scores were calculated using the TIDE tool for all cancer samples in differential risk subgroups. Next, the differential analysis was carried out using the Wilcoxon method, and the results were presented by the raincloud plots drawn by ggplot2 package (v 3.3.5).

### Single-cell analysis

To explore the expression levels of the biomarkers in various types of cells, we carried out expression analyses. The expression of biomarkers in each cell subtype was analyzed using the GSE162454 dataset from the TISCH database (http://tisch.comp-genomics.org/home). The results were presented by heatmaps and violin plots.

### Construction of biomarker-drug interaction network

The drug sensitivity was assessed for each sample in the TCGA-osteosarcoma dataset according to Genomics of Drug Sensitivity in Cancer (GDSC) databases (http://cancerrxgene.org). Next, the half maximal inhibitory concentration (IC50) of drugs were compared within risk groups using Wilcoxon test and box plots of the results were drawn. Next, the correlation between the risk score and the difference drugs (top 2) was calculated using the Spearman method.

### Molecular docking

Firstly, the Comparative Toxicogenomics Database (CTD) database (http://ctdbase.org) was utilized to predict small molecule drugs associated with biomarkers. Then, the small molecule drugs most related to the corresponding biomarkers were screened based on Reference Count sorted from high to low. Subsequently, the predicted molecular structures of small molecule drugs were downloaded through the PDB database (https://www.rcsb.org/). Next, the AutoDock was used to compute the docking case and select the structure with the lowest binding free energy.

### Expression of biomarkers in osteosarcoma samples

The difference of biomarker expression levels between osteosarcoma samples and normal samples was compared in the GSE99671 dataset using the Wilcoxon test. The results were shown by box plots created by the ggplot2 package (v 3.3.5). Then, ten frozen tissue samples (five osteosarcoma samples and five normal samples) from Shanghai Sixth People’s Hospital Affiliated to Shanghai Jiao Tong University School of Medicine were collected. The samples were lysed with TRIzol reagent and total RNA was isolated following the manufacturer’s instructions. The concentration of RNA was measured with a NanoPhotometer N50. Afterwards, RNA was reverse transcribed into cDNA using the SuperScript IV cDNA synthesis kit (Invitrogen, Carlsbad, CA). The reverse transcription-polymerase chain reaction (RT-qRCP) reaction consisted of 3 µL of reverse transcription product, 5 µL of 2xUniversal Blue SYBR Green qPCR Master Mix, and 1 µL each of forward and reverse primer. All primer sequence information were shown in Table 1. The GAPDH gene served as an internal control, and the relative expression of genes was determined using the 2^−ΔΔCt^ method.

**Table 1:** All primer sequence information

gene	primer sequences
EPYC F	GCTGTGACTGCCCCAACTCT
EPYC R	GCCATCAATCAGCCTGGGAG
PANX3 F	CATCTTCACCTCCGCCACTT
PANX3 R	TTTGTCCCACCGACATAGCC
CD36 F	TGAAGGGTTGAGAGCCTGTG
CD36 F	TGCAGGAGCAGATGCAGAAA
CLDN11 R	CTTGGCACTGTAGCATGTGG
CLDN11 R	CAGCAGCAGTAAAATGGCCG
STOM F	TTAGCGTGGATGGTGTGGTC
STOM R	TCTCAAAGAGGAAATCTGAGAAAGA
GAPDH F	CCCATCACCATCTTCCAGG
GAPDH R	CATCACGCCACAGTTTCCC

### Immunohistochemical staining

Tumor tissue slides of osteosarcoma were obtained from Shanghai Sixth People’s Hospital Affiliated to Shanghai Jiao Tong University School of Medicine, which was approved by the Shanghai Sixth People’s Hospital Ethics Committee (Approval No:2021 − 161). The antibodies used for immunohistochemistry were rabbit anti-CD36 (Abcam, cat#ab133625, at the dilution of 1:100,), rabbit anti-Stomatic (Proteintech, cat#12046-1-AP, at the dilution of 1:200), rabbit anti-Pannexin-3 (Affinity, cat#DF7721, at the dilution of 1:50 − 1:200), rabbit polyclonal antibody to Claudin 11(Affinity, cat#AF5364, at the dilution of 1:50 − 1:200), rabbit anti-epiphycan(Abcam, cat#122449, at the dilution of 1:200–1:500). These antibodies were processed and stained according to manufacturers’ protocols. The images were imported to ImageJ software for 8-bit digitization. At 100×magnification, 10 square areas(50 × 50 μm) at the image were randomly selected, and grayscale values were measured using ImageJ software. Mean grayscale values were calculated for each sample. A higher brightness indicated more positive. Both the group and the specimen information were concealed. We scored expression in accordance with the intensity (0, no staining; 1, weak staining; 2, moderate staining; 3, strong staining), and the percentage of cancer cells that were stained (0, none stained; 1, < 10% stained;2, 10–50% stained; 3, > 50% stained; 4, > 75% of all of the cancer cells stained). If the product of multi-plying staining intensity by the percentage of positively stained cancer cells was ≥ 2, it was regarded as positive (+). The percentage area of staining was quantified using ImageJ. Cohen’s kappa coefficient was utilized to assess inter-rater agreement, ensuring the reliability of the scoring. Immunohistochemical (IHC) scoring is vital for predicting patient survival, evaluating tumor pathology grading, and forecasting patients’ responses to specific treatment regimens, highlighting its broad applicability and significant clinical value in the field of oncology.

### Statistical analysis

All bioinformatics analyses were carried out in R software (v 4.2.2). The data of different groups were compared by Wilcoxon test and the results were corrected for Benjamini-Hochberg process (FDR). *P* < 0.05 was considered statistically significant.

## Results

### Identification of DEGs and key module genes

**Fig. 1:**
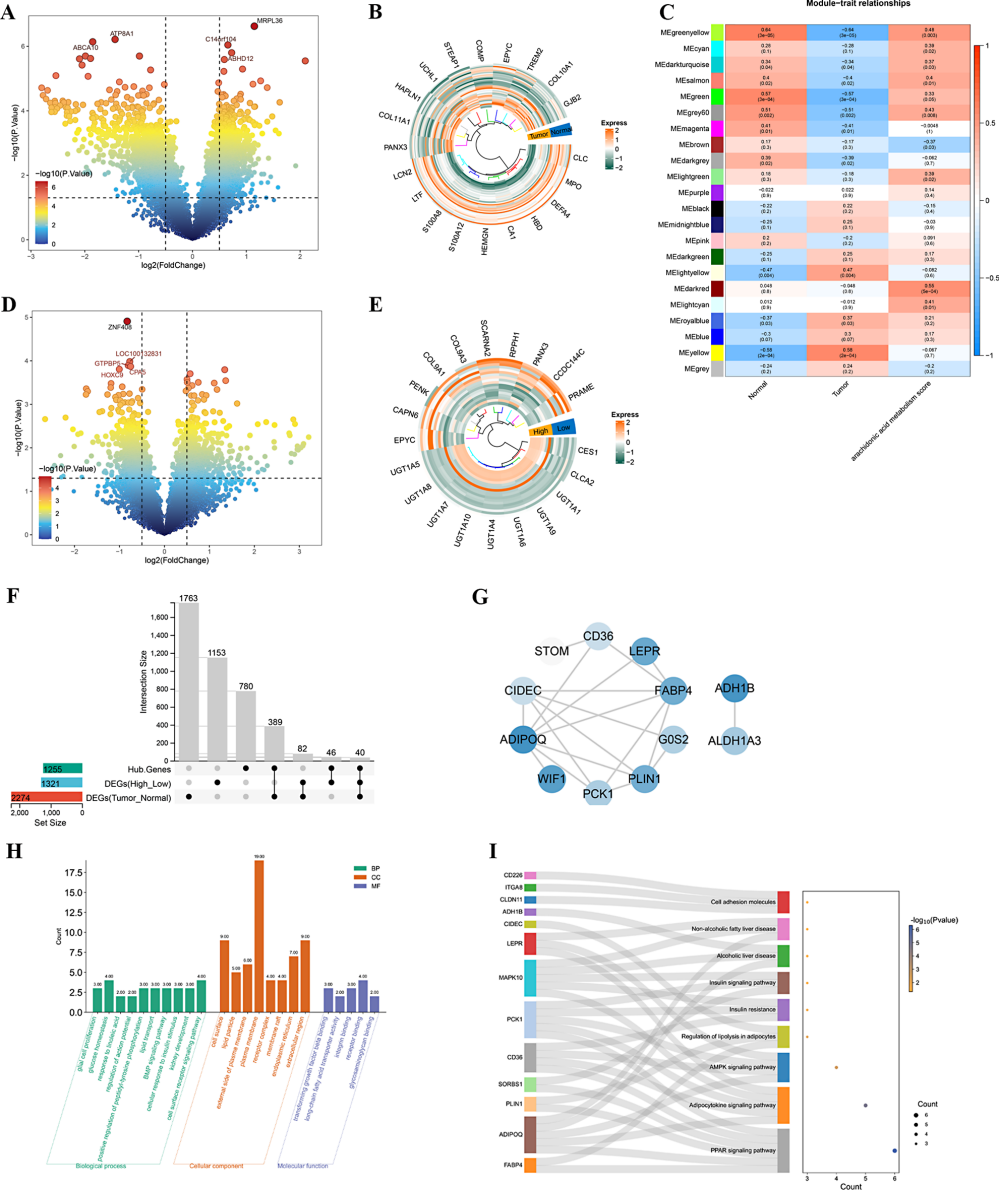
Identification and functional characteristics of differentially expressed genres. (**A**) Volcano map indicate DEG (osteosarcoma vs. control). The blue to orange colours indicate increasingly significant *P* values. Each dot in the graph represents a gene. The top left and top right region genes indicate that they are differentially expressed genes. (**B**) Heatmap of samples in normal and osteosarcoma samples. Yellow for high expression, green for low expression. The darker the color, the greater the expression. (**C**) WGCNA analysis was carried out to find gene s related to AAMRGs scores and osteosarcoma. Red represents positive correlation and blue represents negative correlation. The darker the color, the stronger the correlation. (**D**) Volcano map indicate DE-AAMRGs. The blue to orange colours indicate increasingly significant *P* values. Each dot in the graph represents a gene. The top left and top right region genes indicate that they are differentially expressed genes. (**E**) Heatmap of samples in DE-AAMRGs. Yellow for high expression, green for low expression. The darker the color, the greater the expression. (**F**) Venn diagram of intersecting genes. (**G**) Protein-protein interaction network diagram. (**H**) GO enrichment analyses of intersecting genes. The horizontal axis and vertical axis represent the gene number and term, respectively. Green for BP, orange for CC, purple for MF. (**I**) Venn diagram of intersecting genes enriched in KEGG pathways. Blue indicates a smaller *P* value and orange indicates a larger *P* value. The bluer the color, the smaller the *P* value

In total, 2,274 DEGs between the osteosarcoma and normal group were gained, including 1014 upregulated and 1260 downregulated DEGs (Fig. 1A, Supplementary Table 1). Then, the heat map displays the expression levels of the top 10 upregulated (*PANX3, HAPLN1, UCHL1, STEAP1, COMP, EPYC, TREM2, COL10A, GJB2 and COL11A1*) and downregulated (*LCN2, S100A8, S100A12, HEMGN, CA1, HBD, DEFA4, MPO, CLC and LTF*) genes in osteosarcoma group compared with normal group (Fig. 1B). We performed WGCNA analysis using osteosarcoma samples, normal samples, and arachidonic acid metabolism scores as traits. Furthermore, we successfully constructed 22 modules. Among these, MEdarkturquoise, Mesalmon, Megrey6 and Megreenyellow (|cor| > 0.3, *P* < 0.05) were correlated with osteosarcoma samples, normal samples, and arachidonic acid metabolism scores (Fig. 1C). A total of 1255 Hub genes were obtained by combining above four key modules.

### Acquisition of intersecting genes

Totally, 1,321 DE-AAMRGs between the differential risk subgroups were gained (510 up-regulated and 811 down-regulated) (Fig. 1D, Supplementary Table 2). The heat map displays downregulated genes (*UGT1A5, UGT1A8, UGT1A7, UGT1A10, UGT1A4, UGT1A6, UGT1A9, UG T1A1, CLCA2 and CES1*) in the low-score group, and upregulated genes (*EPYC, CAPN6, PENK, COL9A1, COL9A3, SCARNA2, RPPH1, PANX3, CCDC144C and PRAME*) in the high-score group (Fig. 1E). By intersecting hub genes with differential genes between the osteosarcoma and normal groups, as well as between the high and low score groups, we ultimately identified 40 intersecting genes (Fig. 1F, Supplementary Table 3).

### Functional enrichment of intersecting genes

The PPI network was used to explore the association between the intersection genes, constructed by STRING based on the 40 intersecting genes, comprises 19 nodes and 19 edges. This network analysis revealed potential interactions among these 40 genes (Fig. 1G). To further elucidate the functional characteristics of DE-AAMRGs, we conducted gene functional enrichment analysis using DAVID. Consequently, the results of the enrichment analysis indicated that the intersecting genes implicated 41 GO entries and 9 KEGG pathways. The intersecting genes were mainly enriched to GO entries such as plasma membrane, extracellular region, cell surface (Fig. 1H). Also, the KEGG pathways showed enrichment in various processes primarily concentrated on PPAR signaling pathway, Adipocytokine signaling pathway and AMPK signaling pathway (Fig. 1I, Supplementary Table 4).

### Biomarkers screening and prognostic prediction model

**Fig. 2:**
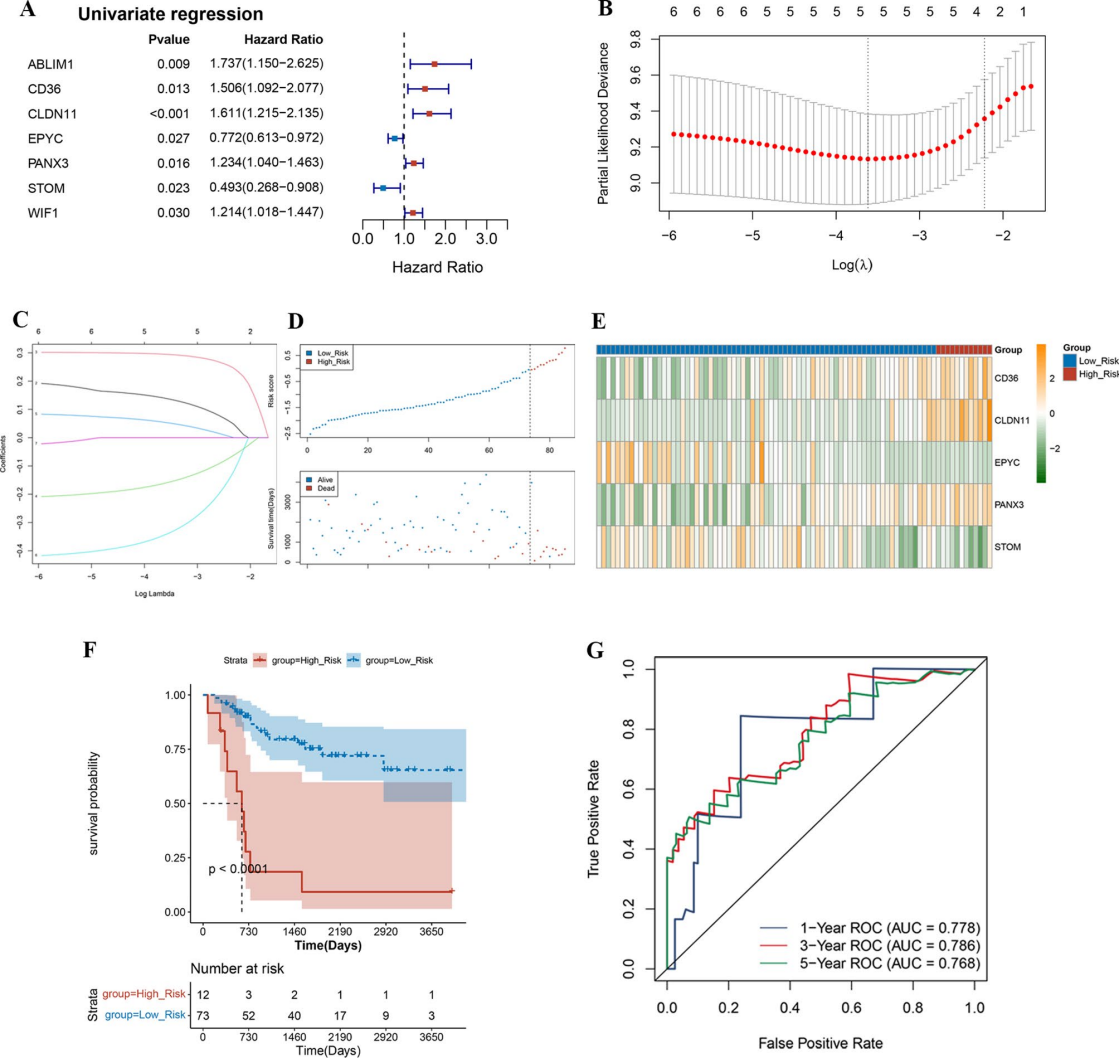
Construction of the prognostic model in the training cohort. **A** Univariate cox proportional hazards regression analysis was used. **B**-**C** LASSO Cox regression method was used to screen candidate genes. **D**-**E** Risk score and survival status of AAMRS in the training set. High expression in orange and low expression in green. **F** Kaplan-Meier analysis of the overall osteosarcoma survival between high-and low-risk groups in the training set. **G** Predict the sensitivity and specificity of 1-, 3-, and 5-years survival according to the risk score in the training set

**Fig. 3:**
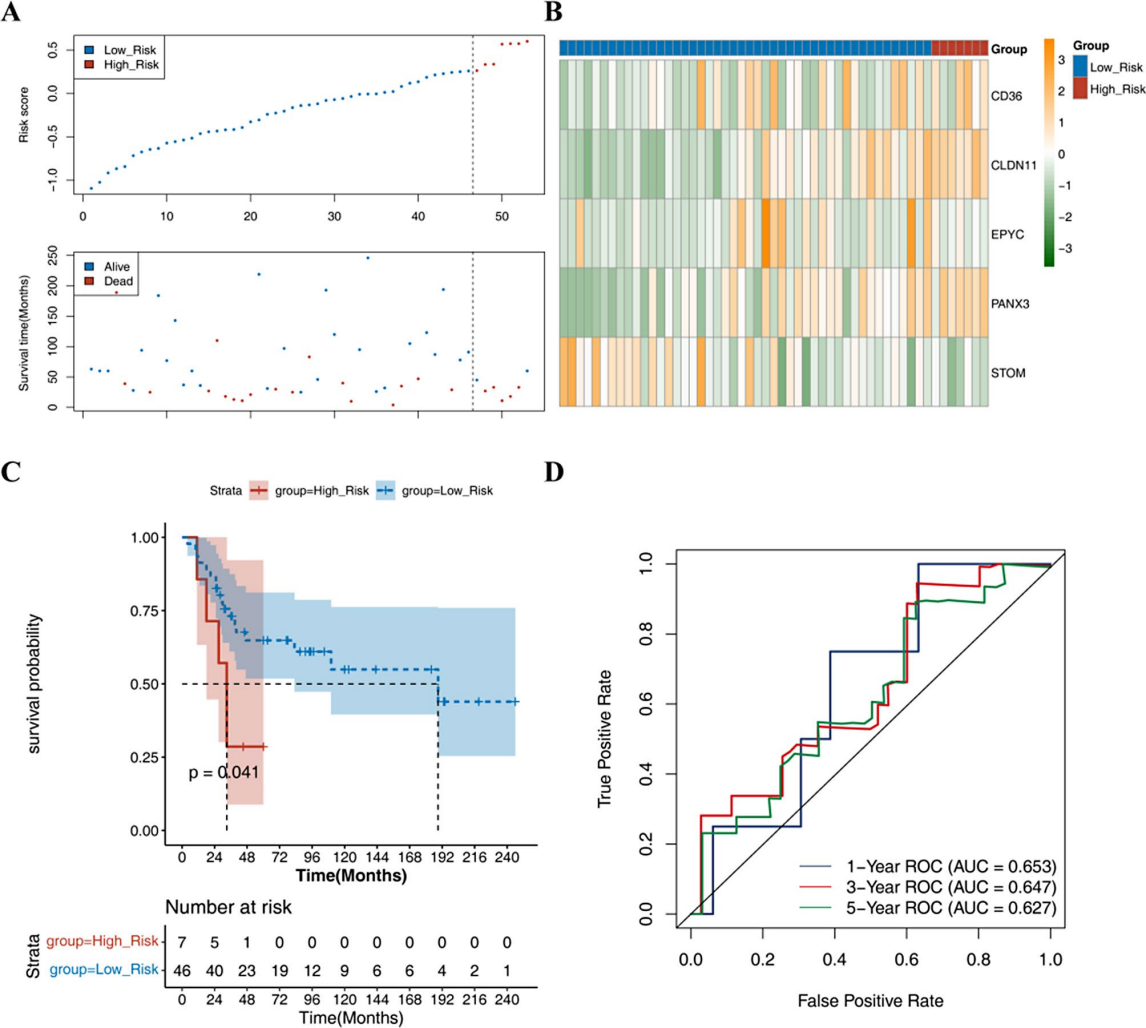
Validation of risk models. **A**-**B** Risk score and survival status of AAMRS in the validation set. High expression in orange and low expression in green. **C** Kaplan-Meier analysis of the overall osteosarcoma survival between high-and low-risk groups in the validation set. **D** Predict the sensitivity and specificity of 1-, 3-, and 5-years survival according to the risk score in the validation set

In order to develop a risk model with the optimal prognostic predictive capacity of these DE-AAMRGs in osteosarcoma patients, we performed univariate Cox analysis and utilized the LASSO method to select a panel of five biomarkers (*CD36*, *CLDN11*, *EPYC*, *PANX3*, and *STOM*) (Fig. 2A and C). According the risk score calculation, patients were classified into high- and low-risk groups on the basis of optimal threshold (-0.04623316) (Fig. 2D). The expression heatmap in(Figure 2E)illustrates the gene expression patterns integral to the construction of the prognostic model. Immediately following, Kaplan-Meier analysis demonstrated that low-risk group had a higher survival rate compared to the high-risk group (Fig. 2F). The ROC curves unveiled a promising predictive performance of the prognostic model, boasting AUC values of 0.778, 0.786, and 0.768 for predicting patients’ osteosarcoma at 1-, 3-, and 5-years, respectively—all exceeding the threshold of 0.76 (Fig. 2G). According the risk score calculation, patients in the validation set were classified into high- and low-risk groups on the basis of optimal threshold (0.2628203) (Fig. 3A). The expression heatmap in Fig. 3B showed the gene expression patterns required to construct the prognostic model. Immediately after that, Kaplan-Meier analysis showed that the survival rate was higher in the low-risk group compared with the high-risk group (Fig. 3C). The ROC curves unveiled a promising predictive performance of the prognostic model, boasting AUC values of 0.653, 0.647, and 0.627 for predicting patients’ osteosarcoma at 1-, 3-, and 5-years, respectively (Fig. 3D).

### Clinical correlation analysis of the prognostic model and nomogram construction

**Fig. 4:**
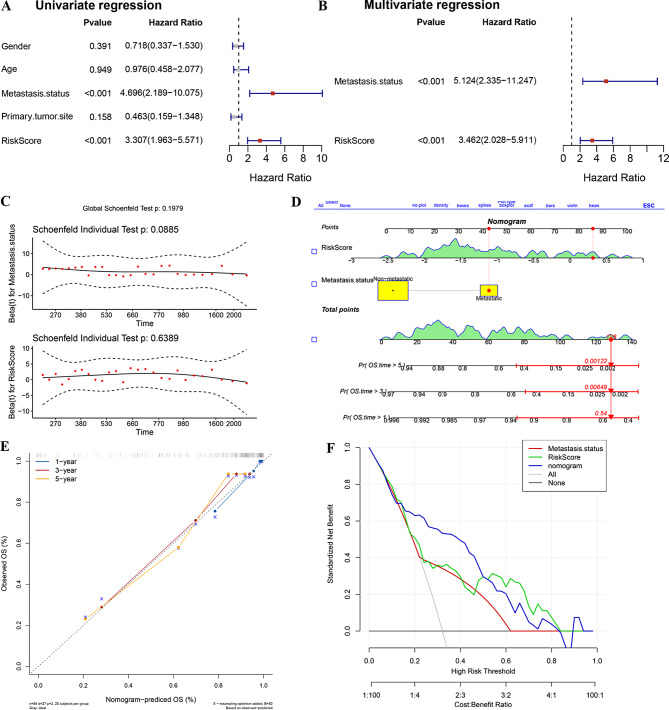
**A**-**D** UniCox and multiCox analysis showed the prognostic value of the risk score. **E** Calibration curve of the prognostic nomogram. **F** DCA curve of the prognostic nomogram

Univariate Cox and multivariate Cox analyses were performed to evaluate the independent prognostic significance of the risk score. The forest plot indicated that two significant factors (Metastasis and risk score) were acquired as an independent risk factor for predicting the prognosis of osteosarcoma patients (Fig. 4A and B). Patients who experienced metastasis exhibited higher risk score in the univariate Cox analysis (satisfied the PH test) and multivariate Cox regression model (Fig. 4C), indicating a correlation between the risk score and increased risk of metastasis and a poorer prognosis. To enhance predictability, the nomogram of prognostic factors points was utilized to predict the 1-, 3-, and 5-year overall survival of osteosarcoma patients (Fig. 4D). According to the results calculated from the total score, a higher score indicates a lower survival rate. The nomogram demonstrated satisfactory accuracy in predicting the 1-, 3-, and 5-year overall survival of osteosarcoma patients, which was validated by the calibration curve (Fig. 4E). As evident from the calibration curve, the slopes at 1-, 3-, and 5-year closely approximate 1, suggesting that the constructed prediction model can serve as an effective tool. The DCA curve also showed higher clinical benefit in the nomogram model (Fig. 4F). Based on the above results, the constructed predictive model can be considered effective, and the clinical benefits of the nomogram model are higher than those of the “Metastasis. status” curve and the “RiskScore” curve.

### Relationship between clinical characteristics and risk scores models

**Fig. 5:**
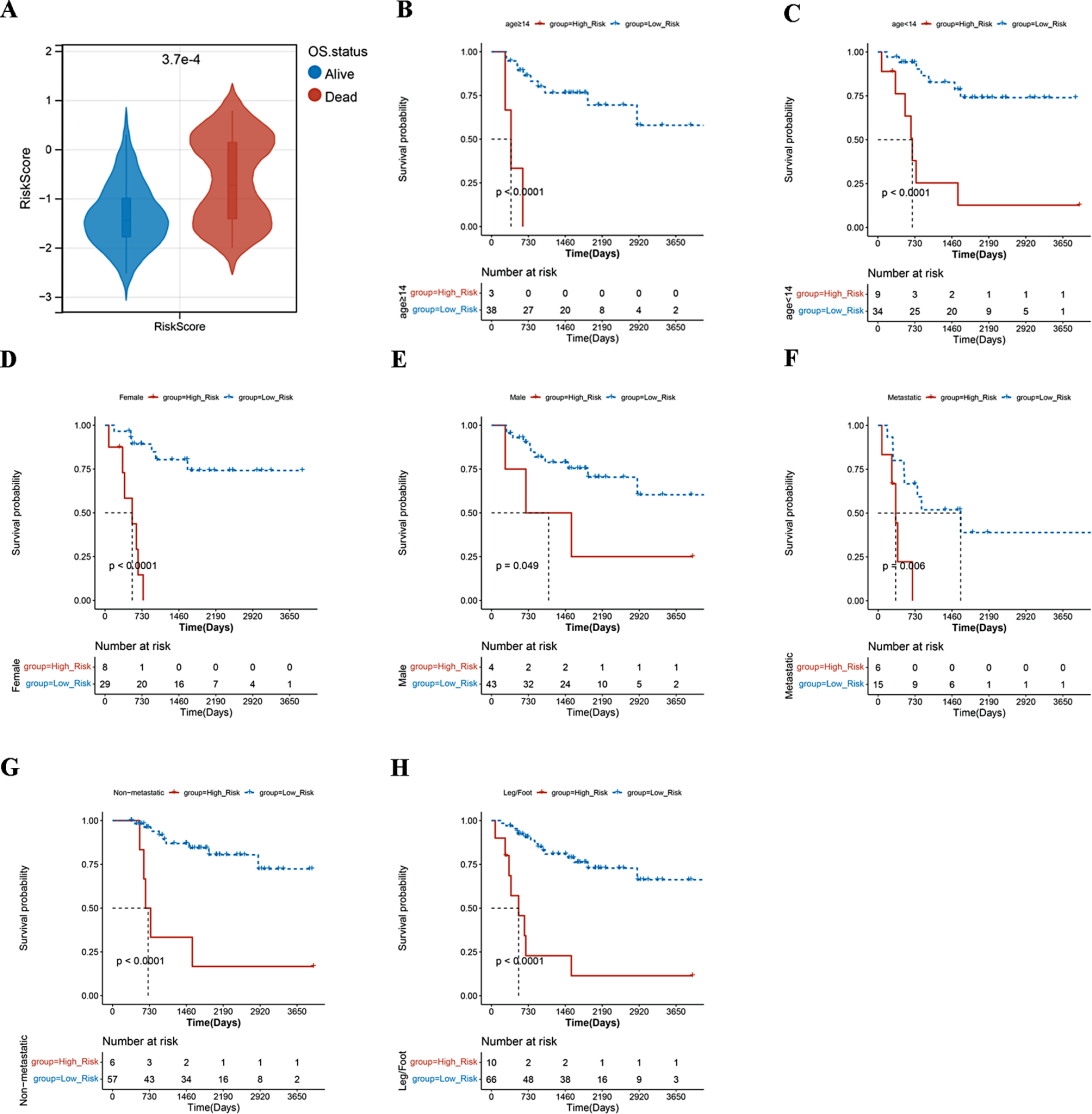
**A** Correlation between risk scores and clinical characteristics. **B**-**H** K-M survival curves of high and low risk groups in different clinical characteristics stratification

Wilcoxon rank sum test was carried out in order to further investigate whether our risk model correlated with the clinical characteristics of osteosarcoma. The risk scores showed no significant variances among the various subgroups of clinical features, except for overall survival status (Fig. 5A, Supplementary Fig. 1). Considering that the prognostically relevant clinical characteristics differed between the two risk groups, we further investigated whether the risk model can be applied to different clinical indicators by using the R package “survival”, including age, gender, primary tumor site, metastasis status. The survival analyses revealed notable differences in survival rates between the two score subgroups concerning age, gender, metastasis status, and primary tumor site in the leg/foot region. In these categories, the survival rate was notably lower in the high-risk group (Fig. 5B and H, Supplementary Fig. 2). The above results suggest that the risk model could either serve as an independent prognostic factor or be integrated with existing clinical indicators.

### GSVA analysis of differential risk subgroups and ssGSEA analysis of biomarkers

**Fig. 6:**
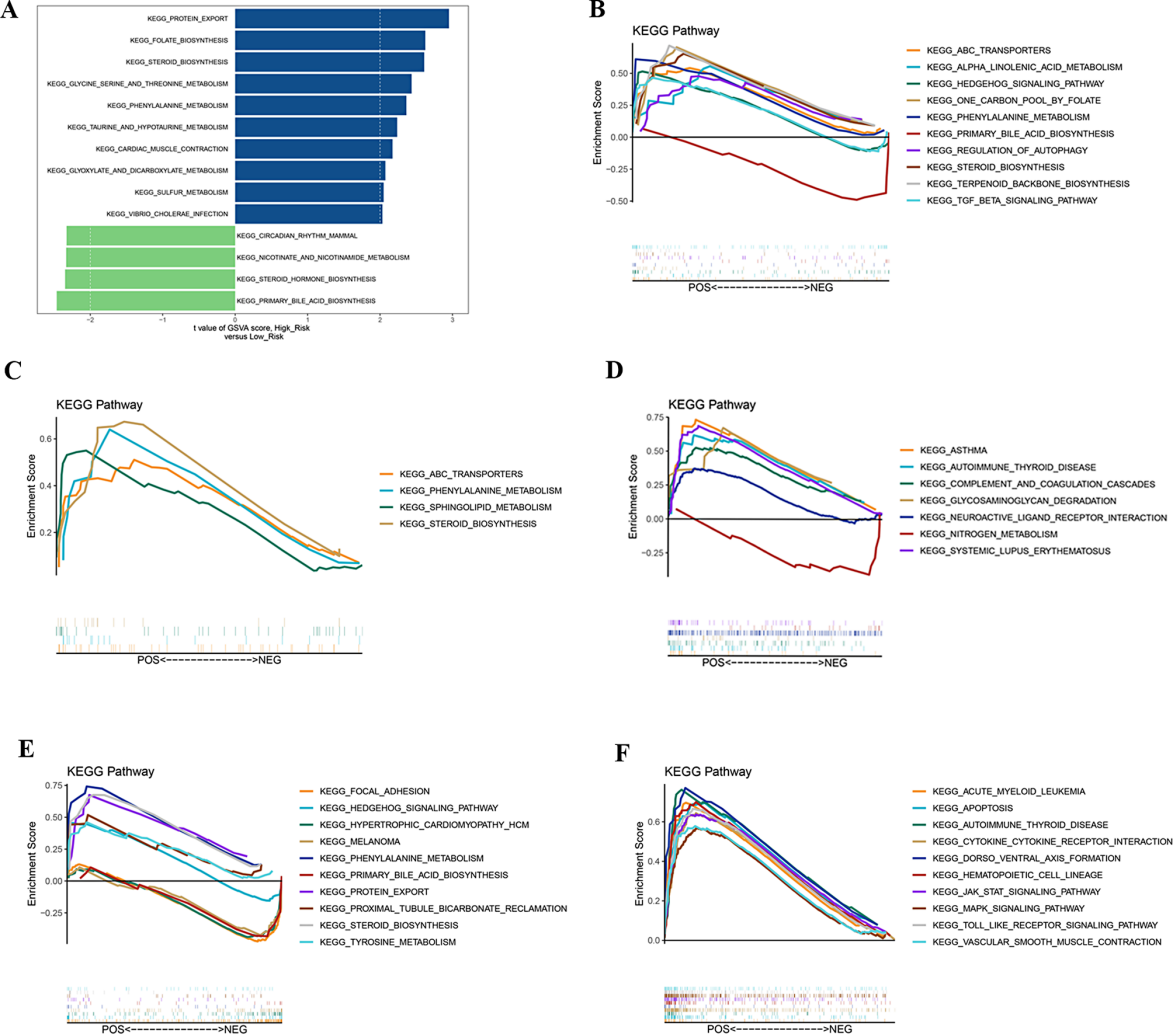
(**A**) Cluster map of differential KEGG pathways in the high and low risk groups. (**B**) Results of KEGG enrichment of CD36 biomarkers. (**C**) Results of KEGG enrichment of CLDN11 biomarkers. (**D**) Results of KEGG enrichment of EYPC biomarkers. (**E**) Results of KEGG enrichment of PANX3 biomarkers. (**F**) Results of STOM enrichment of PANX3 biomarkers

To further validate the function of the risk model in high- and low-risk groups, we performed GSEA pathway enrichment analysis. In the high-risk group, GSVA analysis showed primary enrichment in pathways such as folate biosynthesis and steroid biosynthesis. In contrast, the low-risk group primarily exhibited enrichment in pathways like nicotinate and nicotinamide metabolism and circadian rhythm mammal (Fig. 6A, Supplementary Table 5). The functional enrichment results revealed that CD36 and CLDN11 were mainly enriched in KEGG pathways such as phenylalanine metabolism, steroid biosynthesis (Fig. 6B and C, Supplementary Tables 6–7). EPYC showed significant enrichment in pathways related to neuroactive ligand receptor interaction (Fig. 6D, Supplementary Table 8). PANX3 exhibited enrichment in pathways such as the hedgehog signaling pathway and phenylalanine metabolism (Fig. 6E, Supplementary Table 9), while STOM showed enrichment in pathways like cytokine-cytokine receptor interaction (Fig. 6F, Supplementary Table 10).

### Relationship between risk score model and immune infiltration

**Fig. 7:**
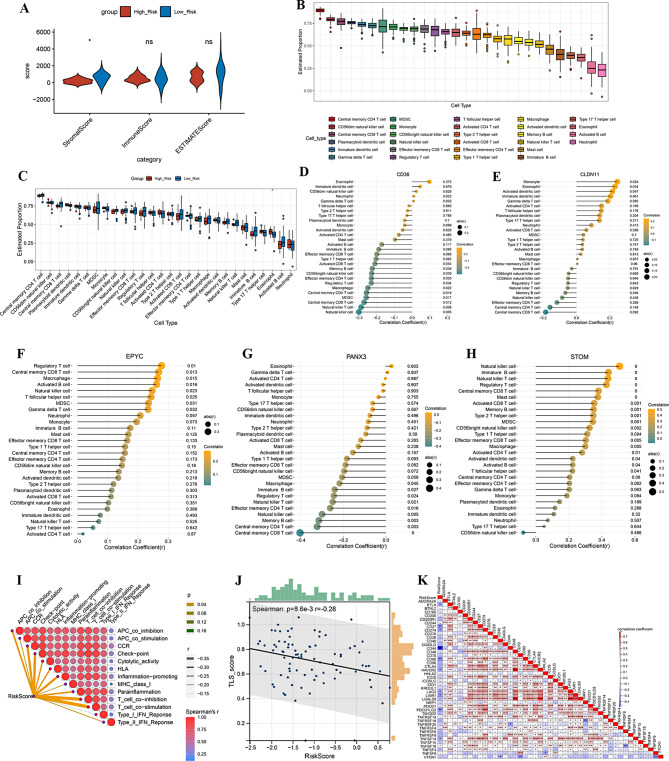
(**A**) The relationship between the risk signature and Stromal Score, Immune Score and ESTIMATE Score. (**B**) 28 immune cell infiltration levels. (**C**) Analysis of the immune infiltration degrees in both groups regarding 28 immune cell. **D**-**H**. Correlations between genes and immune cell infiltration levels involved in the prognostic model. Orange indicates positive correlation, green indicates negative correlation. **I**. Correlations between risk score and immune function. Redder means larger correlation coefficient, greener means smaller *P* value. **J** Correlations between risk score and TLS score. **K** The correlation heatmap between risk score and immune checkpoint. Red represents positive correlation, blue represents negative correlation

To compare the differences in immune scores, stromal scores, and ESTIMATE scores between the high-risk and low-risk groups, we used the ESTIMATE algorithm to calculate these scores for both groups. We then compared the scores between the high-risk and low-risk groups. Our analysis demonstrated that the risk score has significant differences relevant with stromal cell scores between the high- and low-risk groups (Fig. 7A), which suggests that the risk score may indicate increased stromal activity within the tumor-microenvironment. The bars displayed the percentage of the 28 immune cells in each sample (Fig. 7B). Eosinophils, one type of immune cell, exhibited a significant difference between the two risk subgroups (Fig. 7C). Correlation analysis revealed the strongest positive correlation between STOM and natural killer cells, and the strongest negative correlation between PANX3 and central memory CD8 T cells (Fig. 7D and H).

Immunotherapy response related analyses showed significant strongest negative associations between risk scores and T cell co-stimulation, significant weak negative correlations with TLS scores, and strongest negative relationships with the immune checkpoint CD44 (Fig. 7I and K). TIDE analysis showed that only the Exclusion score was different between the risk subgroups (Supplementary Fig. 3).

### Biomarker expression levels in single-cell dataset

**Fig. 8:**
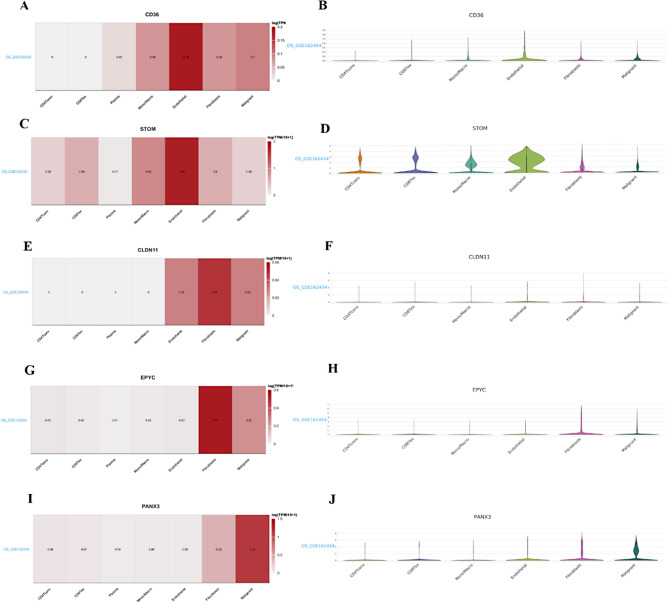
Expression of the five genes included in the prognostic model across each cell subpopulation in the single-cell dataset. Redder colour indicates higher expression

Further investigation into the expression levels of biomarkers in each cell subtype showed that in Endothelial cells, CD36 and STOM exhibited the highest expression levels. In Fibroblasts, CLDN11 and EPYC showed the highest expression levels, while in Malignant cells, PANX3 demonstrated the highest expression (Fig. 8A and J).

### Prediction of biomarker-related drugs

**Fig. 9:**
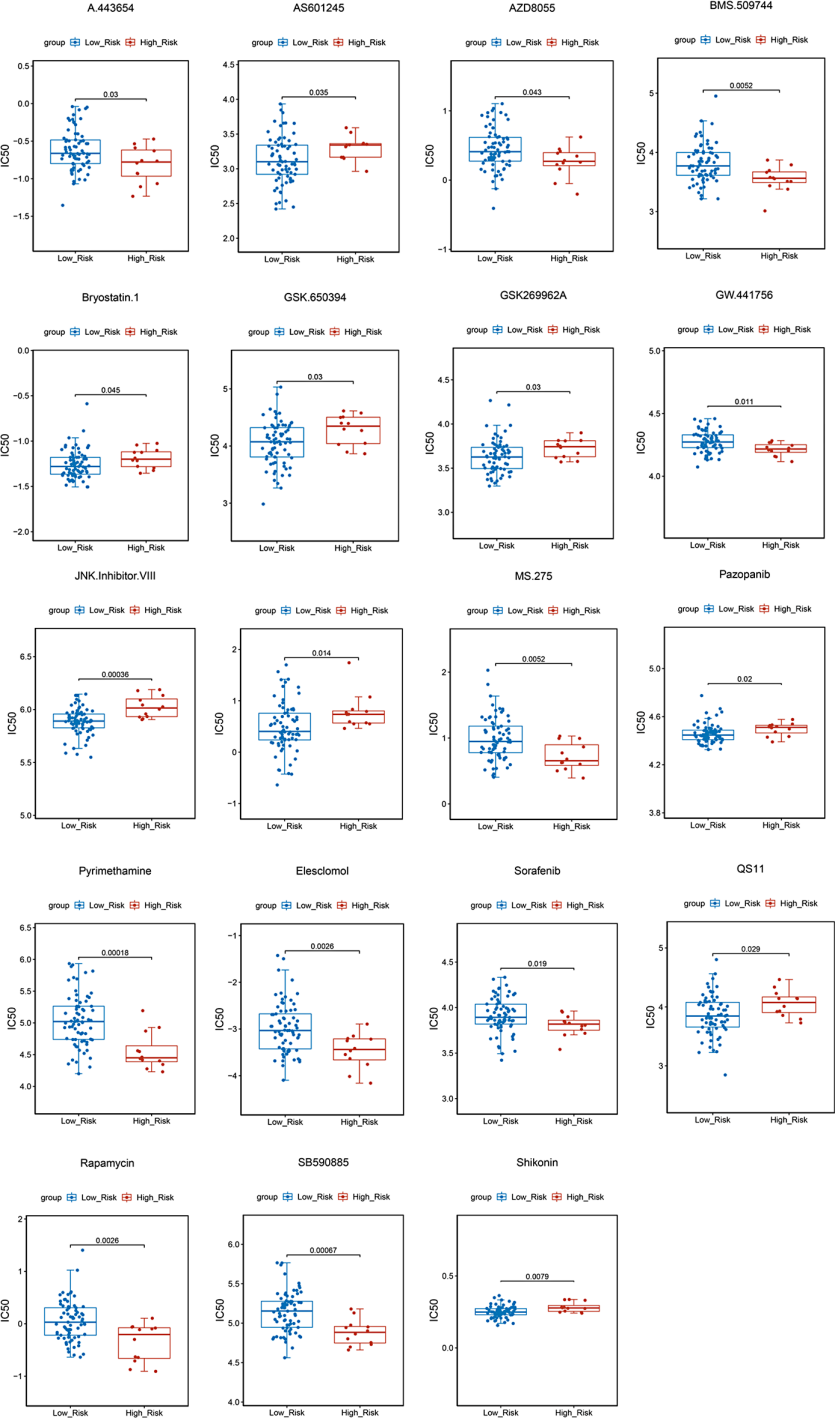
Relationships between risk score and drug sensitivity

**Fig. 10:**
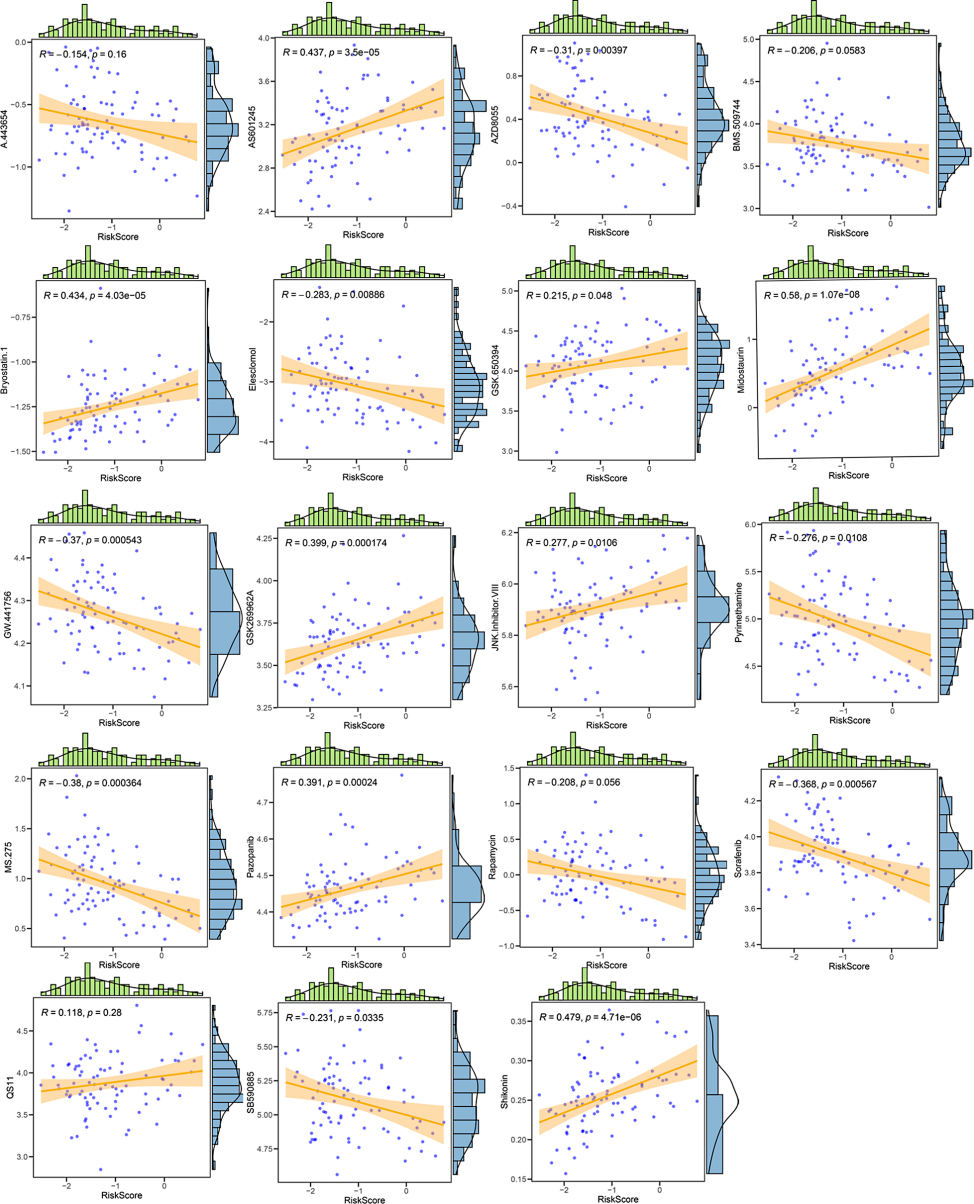
Correlation analysis between risk score and drug sensitivity

Drug sensitivity analysis revealed significant differences in sensitivities to 19 drugs (A.443654, GW.441756, AZD8055, JNK.Inhibitor.VIII, etc.) between the two different risk groups (Fig. 9). Correlation analysis indicated a significant negative association between the risk score and Pyrimethamine, as well as a significant positive correlation with JNK.Inhibitor.VIII (Fig. 10).

### Molecular docking of biomarkers

**Fig. 11:**
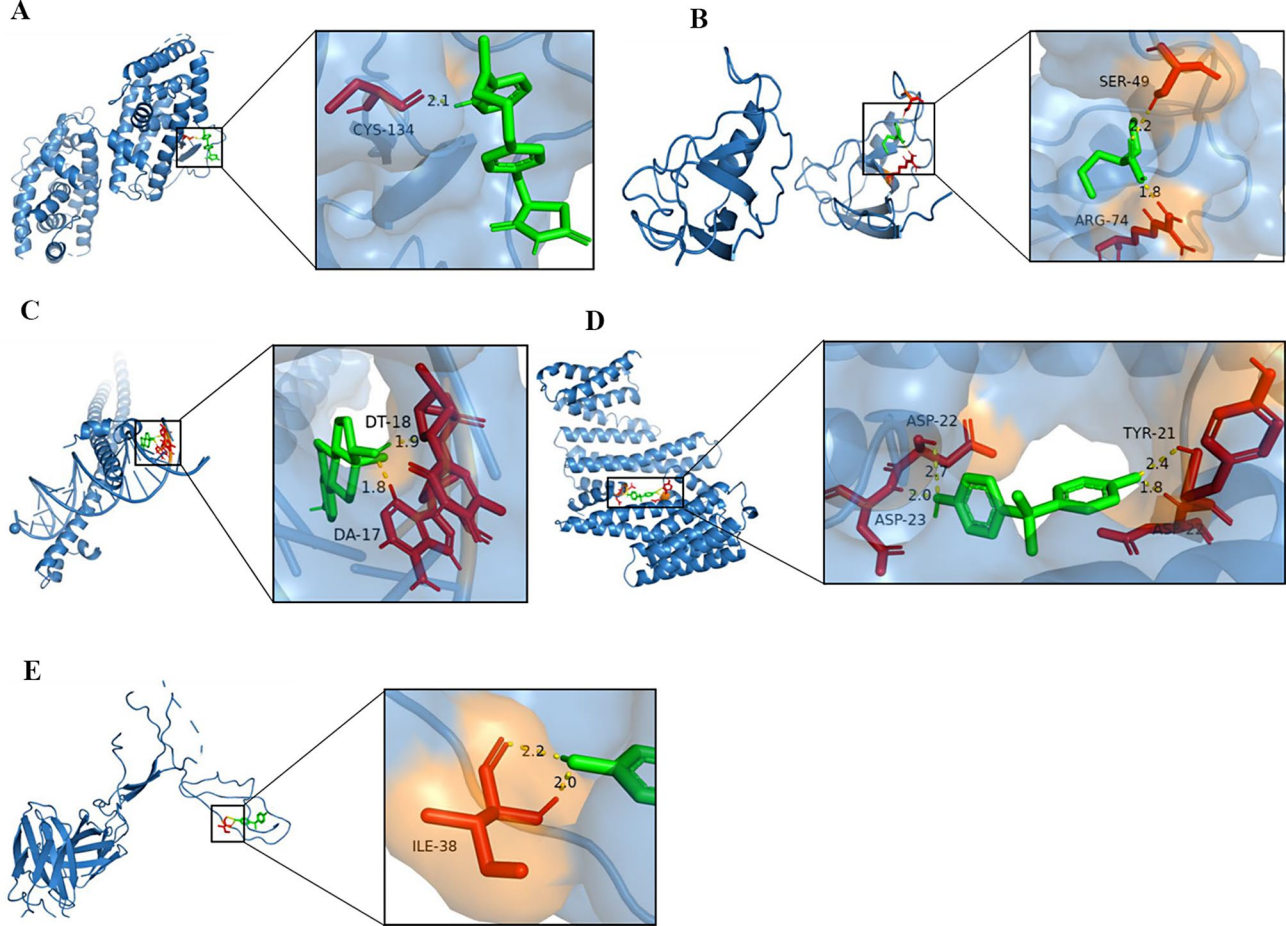
(**A**) CD36-Rosiglitazone molecular docking result; (**B**) CLDN11_Valproic Acid molecular docking results; (**C**) EPYC-Tetrachlorodibenzodioxin molecule docking results; (**D**) PANX3_bisphenol A molecular docking result; (**E**) STOM-bisphenol A molecular docking result

The small molecule drugs most relevant to the corresponding biomarkers were screened as shown in Table 2. The molecular docking was performed for CD36 (PDB ID: 6kz5) and Rosiglitazone (docking score = -2.72 kcal / mol), CLDN11 (PDB ID: 4yyx) and Valproic Acid (docking score = -3.33 kcal / mol), EPYC (PDB ID: 2dg8) and Tetrachlorodibenzodioxin (docking score = -6.21 kcal / mol), PANX3 (PDB ID: 8eq8) and bisphenol A (docking score = -4.37 kcal / mol), and STOM (PDB ID: 4fvf) and bisphenol A (docking score = -4.73 kcal / mol) (Fig. 11).

**Table 2:** The small molecule drugs associated with biomarkers

gene	Chemical Name	Chemical ID	CAS RN	Interaction	Interaction Actions	Reference Count	Organism Count
CD36	Rosiglitazone	D000077154		Rosiglitazone results in increased expression of CD36 mRNA	increases expression	23	4
CLDN11	Valproic Acid	D014635	99-66-1	Valproic Acid results in increased expression of CLDN11 mRNA	increases expression	7	1
EPYC	Tetrachlorodibenzodioxin	D013749	1746-01-6	Tetrachlorodibenzodioxin results in increased expression of EPYC mRNA	increases expression	4	2
PANX3	bisphenol A	C006780	1980/5/7	bisphenol A results in increased methylation of PANX3 gene	increases methylation	1	1
STOM	bisphenol A	C006780	1980/5/7	bisphenol A results in decreased expression of STOM mRNA	decreases expression	4	1

### Biomarker expression analysis of prognostic genes by qRT-PCR and immunohistochemistry

**Fig. 12:**
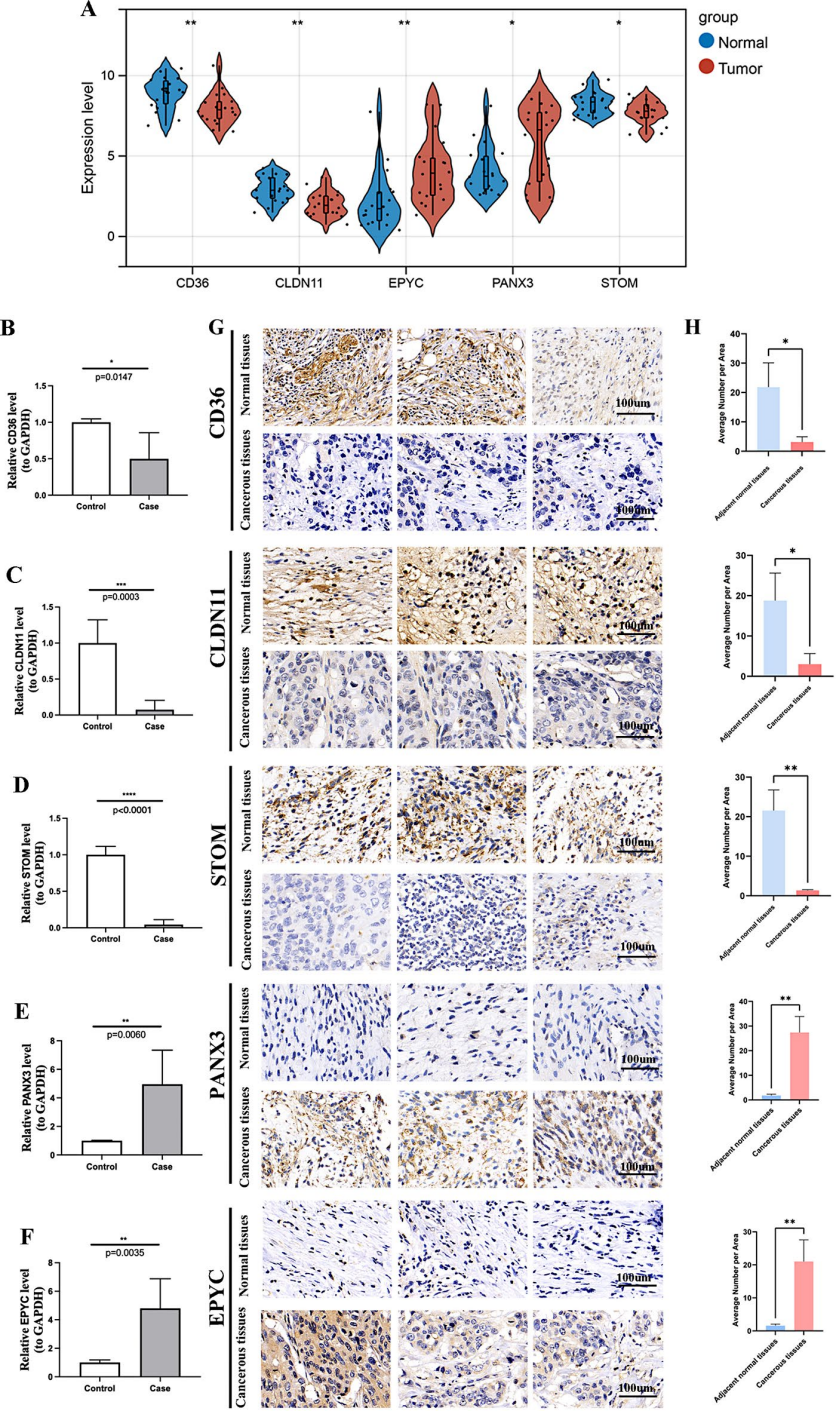
Validation of 5 selected gene expression in osteosarcoma samples was performed by qRT-PCR and Immunohistochemical analysis. (**A**) Expression of these DEGs was normalized against GAPDH expression, EPYC and PANX3 were highly expressed in osteosarcoma cancer. (**B**)The expression level of CD36 is lower in osteosarcoma tissues; (**C**)The expression level of CLDN11is lower in osteosarcoma tissues; (**D**) The expression level of STOM is lower in osteosarcoma tissues; (**E**)The expression level of PANX3 is higher in osteosarcoma tissues; (**F**) The expression level of EPYC is higher in osteosarcoma tissues; (**G**) Immunohistochemical analysis of prognostic genes at osteosarcoma cancerous tissues and normal tissues. The brown staining areas show the positive expression; (**H**) Average number per Area(%). The statistical significance of differences was calculated by the T-test

The differential analysis showed that in the osteosarcoma group, the expression of EPYC and PANX3 was obviously elevated, whereas the expression of CD36, CLDN11 and STOM was markedly decreased (Fig. 12A). To validate the expression of biomarkers, five pairs of osteosarcoma and normal frozen tissues samples were collected and RT-qPCR was performed to elucidate the changes in expression of biomarkers in the osteosarcoma and normal groups. The expression levels of EPYC and PANX3 were significantly higher in osteosarcoma samples than in normal groups, while the trends of CD36, CLDN11 and STOM were reversed, which was consistent with results from public database (Fig. 12B and F).We further confirmed the levels of the above prognostic genes in osteosarcoma tissue samples and adjacent non-tumorous tissue by immunohistochemistry. The IHC analysis displayed that osteosarcoma tissues express significantly higher levels of EPYC and PANX3 compared to adjacent non-tumorous tissues. In addition, CD36, CLDN11 and STOM were found to have lower expression between osteosarcoma tissue samples compared to para-carcinoma tissue. (Figure 12G and H)

## Discussion

In the current study, we aimed to elucidate the molecular mechanisms underlying the prognostic relevance of AAMRGS in predicting survival outcomes in osteosarcoma. By analyzing osteosarcoma data from the TCGA and GEO databases, We identified 40 intersecting genes associated with survival prognosis. The WGCNA played an important role in revealing key genes involved in the tumor development process, as demonstrated by the studies of Xiaoqi Zhang et al. [19]and Xi Xiao et al. [20]. Our subsequent investigation explored the combined influence of multiple gene biomarkers on the overall survival prognosis of osteosarcoma patients. Further analysis revealed five biomarkers: CD36, CLDN11, EPYC, PANX3, and STOM as a novel AA metabolic prognostic signature. Validation of gene expression through public databases and RT-qPCR results showed significant upregulation of EPYC and PANX3 in osteosarcoma samples, while CD36, CLDN11, and STOM were significantly downregulated. Additionally, IHC analysis was performed to corroborate our RT-qPCR findings and detect protein expression in osteosarcoma tissues. The results from the prognostic tests were consistent with those from the PCR analysis. The pannexin family, comprising PANX1, PANX2, and PANX3, forms glycoprotein channels in the cell membrane [21], influencing various biological processes like keratinocyte differentiation, apoptosis, monocyte recruitment, osteoblast differentiation, and carcinogenesis. Particularly, PANX3, implicated in osteoblast differentiation and osteosarcoma, is dysregulated in carcinogenesis, showing upregulation in axillary sweat gland carcinoma and osteosarcoma, correlating with osteosarcoma prognosis [22, 23]. Whole transcriptome analysis has further identified an up-regulation of in osteosarcoma tissues. A previous study also confirmed that overexpression of PANX3 significantly promoted the proliferation, invasion for osteosarcoma cells and attenuated the antitumor efficacy of mir-431-5p mimics in osteosarcoma cells [24]. Recent research indicates a significant correlation between PANX3 and osteosarcoma. Its overexpression not only unveils new insights into the development of osteosarcoma but also serves as a key element of its molecular characteristics, offering new perspectives and potential therapeutic targets for research [25]. This study concurs with previous research by finding significant overexpression of PANX3 in cancer samples. The epiphycan (EPYC), also known as dermatan sulfate proteoglycan 3 (DSPG3), is a member of the limited family of leucine-rich repeat proteoglycans [26]. EPYC has been implicated in critical roles across a spectrum of human diseases, including osteoarthritis [27], high myopia [28], rheumatoid arthritis [24], among others. Nonetheless, the specific function of EPYC within the context of tumor biology remains to be elucidated. Recent studies have reported EPYC as a significant independent prognostic factor in ovarian [29] and prostate cancer [30]. Moreover, previous research has shown that EPYC can promote the proliferation of pancreatic cancer through the PI3K-AKT signaling pathway, highlighting its potential as a prognostic biomarker for the disease [31]. However, the research concerning the involvement of EPYC in osteosarcoma is non-existent. In our study, we found that EPYC plays a crucial role in the malignant progression of osteosarcoma and serves as an independent predictor of poor prognosis. Additionally, EPYC expression levels were elevated in osteosarcoma tissues compared to adjacent non-tumorous tissues. Our findings offer a new approach to clinical osteosarcoma management. CD36, a transmembrane glycoprotein, serves vital functions in lipid homeostasis, angiogenesis, immune response, cellular adhesion, and metastasis across different cancers. A previous study highlights that osteosarcoma cells (MG-63) with elevated thrombospondin-1 (TSP-1) levels undergo apoptosis and exhibit inhibited angiogenesis via the endothelial CD36 receptor. This underscores the potential of the TSP-1/CD36/vascular axis as a promising anti-cancer target [32]. Furthermore, evidence suggests that CD36 expression inhibits tumor aggressiveness, and high levels of CD36 expression correlate with extended patient survival and favorable prognoses [33]. However, the literature presents conflicting views on CD36’s role; while downregulation of CD36 mRNA has been reported to reduce osteosarcoma cell proliferation [34], further investigation is needed to clarify its function in osteosarcoma. Recent studies indicate that CD36 plays a significant role in osteosarcoma, acting not only as a key biomarker for prognosis but also as a potential therapeutic target, thereby providing new strategies for personalized treatment of the disease [35]. The roles of PANX3 and CD36 are well established; however, the specific functions of EPYC, CLDN11, and STOM in osteosarcoma remain unclear. This study identifies EPYC, CLDN11, and STOM for the first time as biomarkers associated with the prognosis of osteosarcoma. The findings indicate that EPYC plays a central role in the malignant progression of osteosarcoma and serves as an independent predictor of poor prognosis. Furthermore, EPYC expression levels in osteosarcoma tissue are significantly higher than those in adjacent non-tumor tissue. This discovery paves the way for new clinical treatment strategies for osteosarcoma. Thus, further investigation into the precise functions of EPYC, CLDN11, and STOM is essential.

This study also emphasizes the association of AAMRGs with the PPAR signaling pathway. Previous research has suggested that oridonin, a compound, exerts a substantial proapoptotic effect in both in vitro and in vivo settings by activating PPAR-γ and inhibiting the Nrf2 pathway. This suggests a potential mechanistic pathway for the influence of AAMRGs on osteosarcoma prognosis [36]. Interestingly, our study observed a significant difference in the infiltration levels of eosinophils between the two risk subgroups, the expression level was higher in the high-risk group. Eosinophils originate from myeloid progenitors in the bone marrow and, under the influence of cells within the TME and cytokines, activated eosinophils modulate the behavior of other immune cells, either promoting or inhibiting tumor growth [37]. The precise role of eosinophils in either promoting or inhibiting cancer cells remains a subject of debate. Previous studies have suggested that eosinophils can enhance the response to immune checkpoint blockade in breast cancer by activating CD8 + T cells [38]. Although the function of eosinophils in osteosarcoma is not extensively documented, past research has underscored their significant role in angiogenesis [39]. Notably, our findings confirm a frequent accumulation of eosinophils in the high-risk group, marking the detection of eosinophils in osteosarcoma as a novel discovery meriting further investigation to explore potential correlations with metastasis or prognosis. Intriguingly, our analysis revealed significant differences in eosinophil infiltration between high-risk and low-risk groups. Eosinophils, derived from myeloid progenitors, can exert either positive or negative influences on tumor dynamics based on their interactions within the tumor microenvironment (TME). Despite evidence suggesting a beneficial effect of eosinophils on immune checkpoint blockade response in breast cancer and their involvement in angiogenesis, their role in osteosarcoma necessitates further exploration. The frequent presence of eosinophils in high-risk osteosarcoma cases offers a new avenue for investigation into their potential link to metastasis or prognosis.

Utilizing five identified biomarkers, we developed a risk model that exhibits promising capabilities for predicting the prognosis of osteosarcoma patients. It’s worth noting that our model achieved AUC values of 0.778, 0.786, and 0.768 for 1-, 3-, and 5-year survival, respectively, within the TCGA-osteosarcoma dataset. These values underscore its good performance and reliability in prognostic risk assessment for osteosarcoma patients. In line with our expectations, patients classified into high-risk category presented with poorer prognoses than those categorized as low-risk. Furthermore, through a series of analytic procedures, we identified metastasis status and risk score as independent prognostic factors for osteosarcoma patients. This signifies that the prognostic significance of metastasis status and risk score remains unaffected even when other variables are taken into consideration. In future research, there is an opportunity to enhance clinical assessments of patient prognoses by integrating two key factors, thus improving the accuracy of prognostic evaluations. The integration of these independent prognostic factors into a nomogram notably increased the precision and clinical significance of our model for predicting outcomes in osteosarcoma patients. This underscores its potential significance in the domain of personalized patient care and decision-making. The nomogram we have devised could prove to be a valuable tool for prognostic prediction, with practical implications for the management of osteosarcoma patient care. Similarly, our findings from immune-related and drug sensitivity analyses offer valuable insights, laying the groundwork for tailored and precise treatment strategies for osteosarcoma patients. These discoveries show promise for guiding future research endeavors and assisting clinicians in tailoring therapy plans to suit the distinctive features of each patient’s condition.

This study offers a significant advantage by employing bioinformatics methods to explore prognostic biomarkers linked to osteosarcoma, compare the immune microenvironment of osteosarcoma and normal groups, and investigate pathways or biological functions enriched by these biomarkers. Furthermore, the efficacy of the prognostic model relies on selecting gene combinations that have undergone experimental validation. However, this study has certain limitations, bioinformatics research relies on a variety of tools and algorithms, which may introduce certain biases and limitations that could potentially affect the accuracy of the findings. Although this study identified five potential biomarkers, the in-depth investigation of their underlying mechanisms remains insufficient. Therefore, future research should integrate functional experiments and animal models to more comprehensively elucidate the roles and mechanisms of these biomarkers. Additionally, the practical value and potential of these biomarkers in clinical applications require further exploration and validation to advance their translation into precision medicine.

## Electronic supplementary material

Below is the link to the electronic supplementary material.

Supplementary Material 1

Supplementary Material 2

Supplementary Material 3

Supplementary Material 4

Supplementary Material 5

Supplementary Material 6

Supplementary Material 7

Supplementary Material 8

Supplementary Material 9

Supplementary Material 10

Supplementary Material 11

## Data Availability

All data generated or analyzed during this study are included in this published article and its supplementary information files.

## Abbreviations

OS: Osteosarcoma
AA: Arachidonic acid
AAMRGs: Arachidonic acid metabolism-related genes
TCGA: The Cancer Genome Atlas
GEO: Gene Expression Omnibus
WGCNA: Weighted Gene Co-expression Network Analysis
DEGs: Differentially Expressed Genes
ROC: Receiver Operating Characteristic
TME: The Tumor Microenvironment
cPLA2: Cytosolic Phospholipase A2
COX: Cyclooxygenase, LOX: Lipoxygenase
CYP450: Cytochrome P450
PGs: Prostaglandins
LTs: Leukotrienes
AUC: Area Under Curve
