# Supplementary material for: Role of arachidonic acid metabolism in osteosarcoma prognosis by integrating WGCNA and bioinformatics analysis
Source: BMC Cancer. 2025 Mar 12;25:445. doi: 10.1186/s12885-024-13278-3 (PMC11905593; doi:10.1186/s12885-024-13278-3)
Supplement: Supplementary file 11 — Supplementary Material 11 [file 12885_2024_13278_MOESM11_ESM.docx]

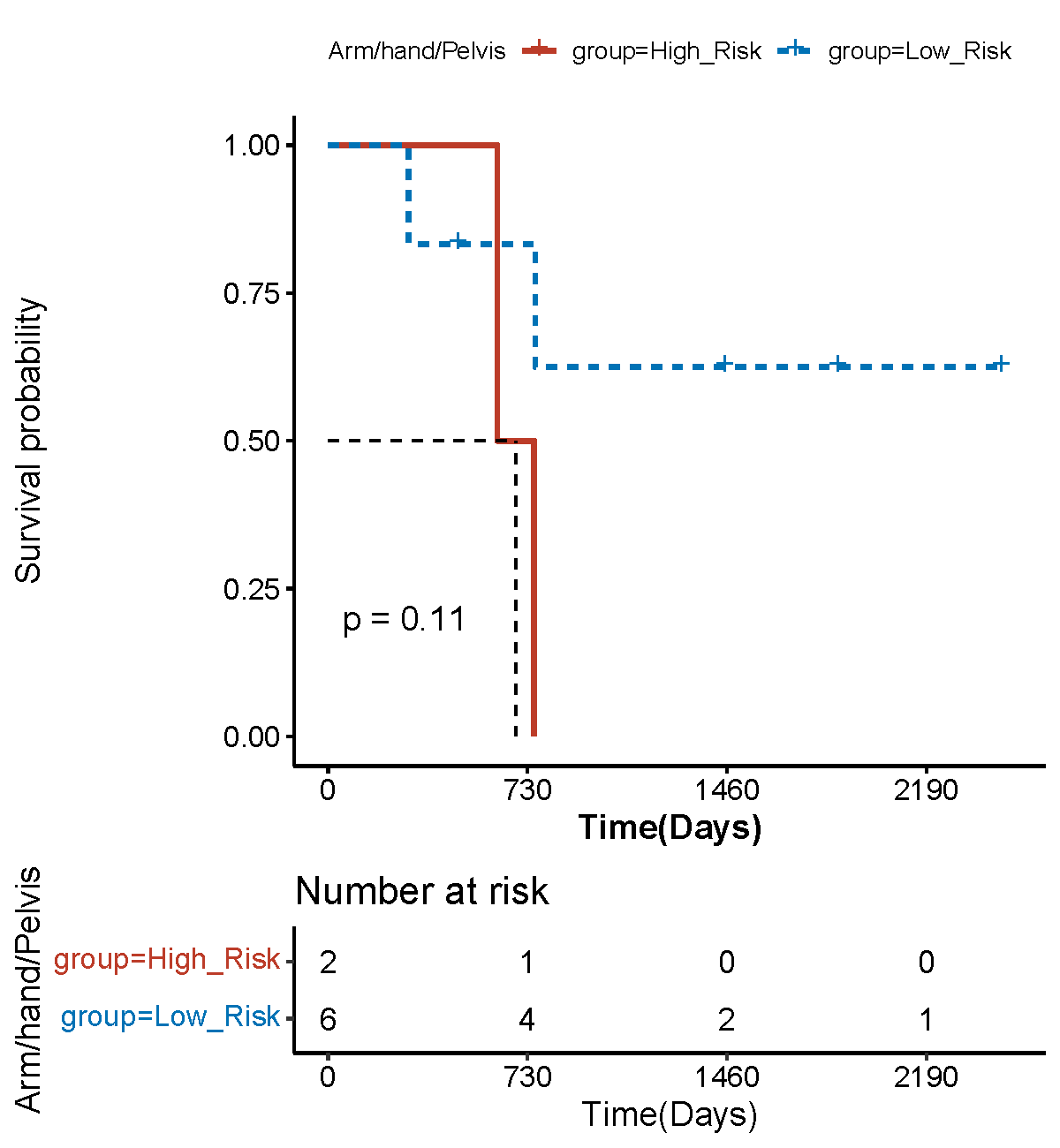


**Supplementary Figure 1.** The K-M survival curve for Arm/hand/Pelvis stratification for high and low risk groups.


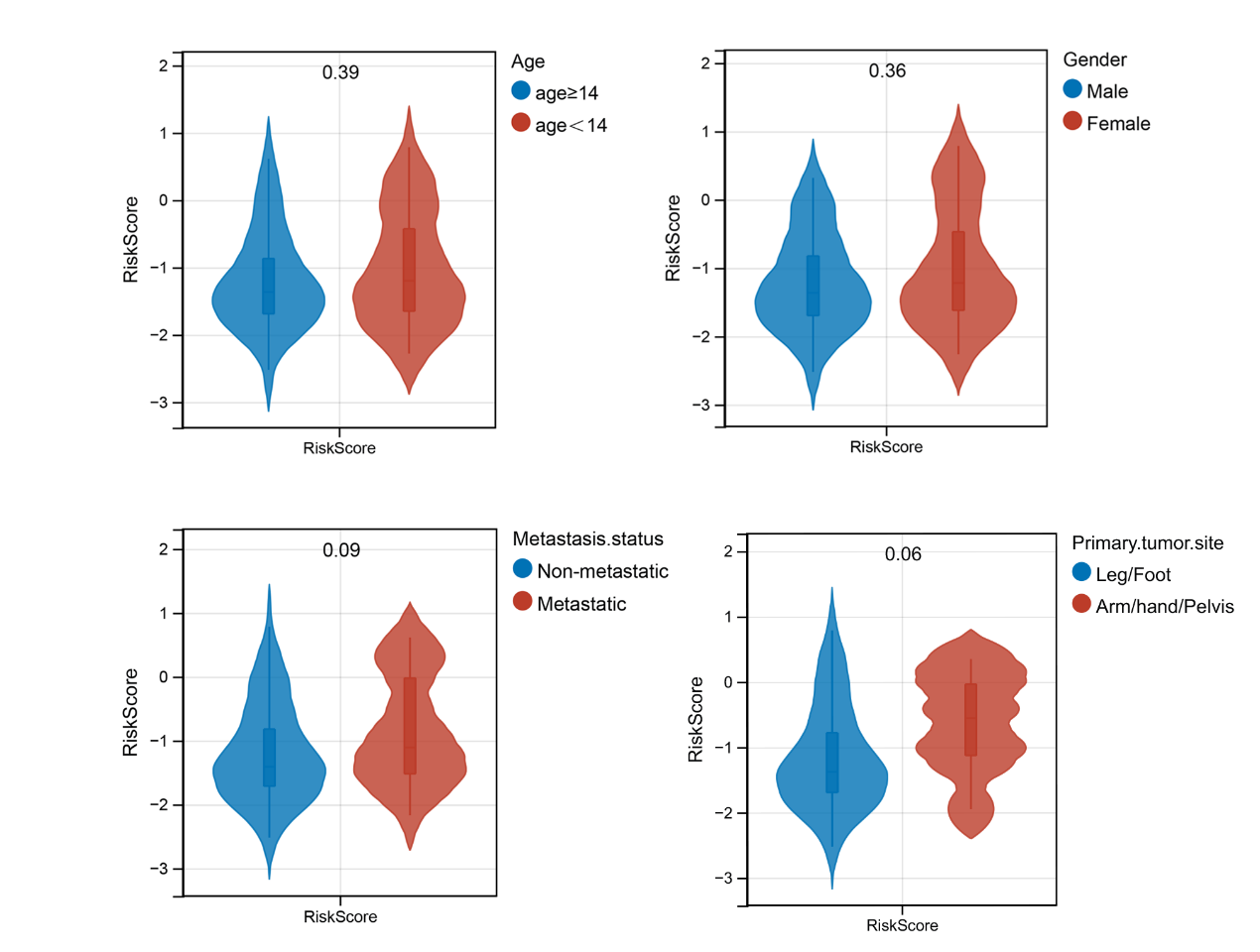


**Supplementary Figure 2.** The violin plot of correlation between risk score and clinical characteristics (age, gender, Metastasis.status, Primary.tumor.site).


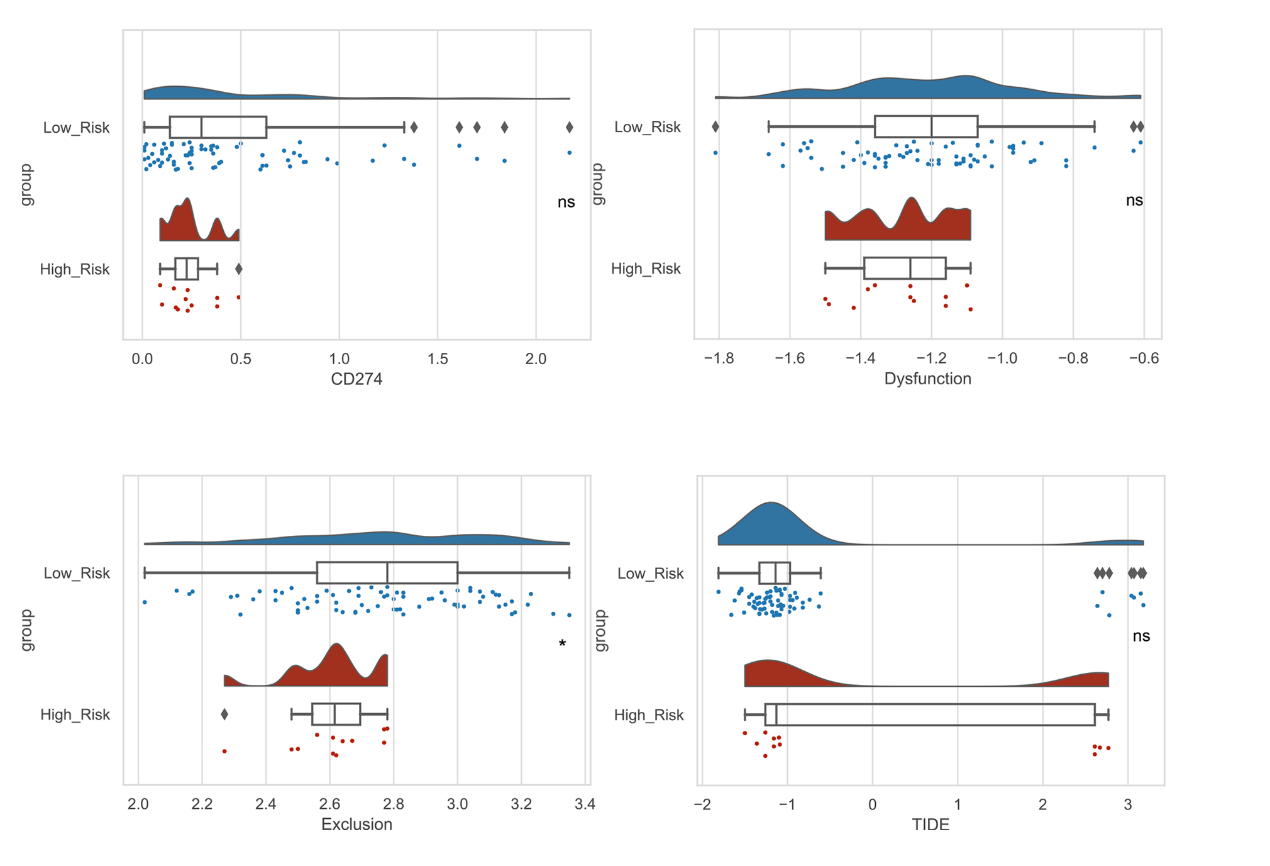


**Supplementary Figure 3.** Cloud and rain plot of scores such as CD274, Dysfunction, Exclusion and TIDE in high and low risk groups. * p < 0.05, ns: p > 0.05.
